# Epidemiology of common infectious diseases before and during the COVID-19 pandemic in Bavaria, Germany, 2016 to 2021: an analysis of routine surveillance data

**DOI:** 10.2807/1560-7917.ES.2023.28.41.2300030

**Published:** 2023-10-12

**Authors:** Sarah van de Berg, Tanja Charles, Achim Dörre, Katharina Katz, Stefanie Böhm

**Affiliations:** 1 Department of Infectious Disease Epidemiology, Postgraduate Training for Applied Epidemiology (PAE), Robert Koch Institute, Berlin, Germany; 2 European Programme for Intervention Epidemiology Training (EPIET), European Centre for Disease Prevention and Control (ECDC), Stockholm, Sweden; 3 Bavarian Health and Food Safety Authority, Munich, Germany

**Keywords:** infectious diseases, surveillance, pandemic, non-pharmaceutical interventions

## Abstract

**Background:**

Unprecedented non-pharmaceutical interventions to control the COVID-19 pandemic also had an effect on other infectious diseases.

**Aim:**

We aimed to determine their impact on transmission and diagnosis of notifiable diseases other than COVID-19 in Bavaria, Germany, in 2020 and 2021.

**Methods:**

We compared weekly cases of 15 notifiable infectious diseases recorded in Bavaria between 1 January 2016 and 31 December 2021 in time series analyses, median age and time-to-diagnosis using Wilcoxon rank sum test and hospitalisation rates using univariable logistic regression during three time periods: pre-pandemic (weeks 1 2016–9 2020), pandemic years 1 (weeks 10–52 2020) and 2 (2021).

**Results:**

Weekly case numbers decreased in pandemic year 1 for all diseases assessed except influenza, Lyme disease and tick-borne encephalitis; markedly for norovirus gastroenteritis (IRR = 0.15; 95% CI: 0.12–0.20) and pertussis (IRR = 0.22; 95% CI: 0.18–0.26). In pandemic year 2, influenza (IRR = 0.04; 95% CI: 0.02–0.09) and pertussis (IRR = 0.11; 95% CI: 0.09–0.14) decreased markedly, but also chickenpox, dengue fever, *Haemophilus influenzae* invasive infection, hepatitis C, legionellosis, noro- and rotavirus gastroenteritis and salmonellosis. For enterohaemorrhagic *Escherichia coli* infections, median age decreased in pandemic years 1 and 2 (4 years, interquartile range (IQR): 1–32 and 3 years, IQR: 1–18 vs 11 years, IQR: 2–42); hospitalisation proportions increased in pandemic year 1 (OR = 1.60; 95% CI: 1.08–2.34).

**Conclusion:**

Reductions for various infectious diseases and changes in case characteristics in 2020 and 2021 indicate reduced transmission of notifiable diseases other than COVID-19 due to interventions and under-detection.

**Key public health message**

**What did you want to address in this study?**

In this study, we investigate if and how the spread and detection of infectious diseases other than COVID-19 changed in Bavaria during the pandemic in 2020 and 2021, when substantial measures were in place to reduce the spread of COVID-19.

**What have we learnt from this study?**

Case numbers of several infectious diseases other than COVID-19 decreased in 2020 and 2021 – both diseases transmitted directly from person to person and diseases transmitted via other routes. This indicates that the measures were effective in reducing also the spread of other infectious diseases. Part of the observed effect may also be caused by the fact that some people did not seek care or were not tested for certain diseases during the pandemic.

**What are the implications of your findings for public health?**

Our findings provide a baseline to help interpret post-pandemic developments in infectious diseases other than COVID-19. We will need to determine if case numbers return to pre-pandemic levels as measures are discontinued and access to healthcare is fully restored or if case numbers will even increase as the level of immune protection may have decreased or diagnoses may have been missed during the pandemic.

## Introduction

The COVID-19 pandemic has had a fundamental impact on health services [1-3] and the epidemiology of other infectious diseases worldwide [4]. Studies from several countries found decreases in case numbers of respiratory diseases such as influenza, pertussis and respiratory syncytial virus (RSV) infection, gastro-intestinal infections such as rotavirus and norovirus infection [4], sexually transmissible diseases [5-7], vector-borne diseases [8,9] and food-borne diseases [10]. With the exception of tick-borne encephalitis (TBE), case numbers for all notifiable diseases decreased significantly in Germany from March until August 2020, compared with the same period in 2016 to 2019 [11]. Non-pharmaceutical interventions (NPIs) implemented to reduce transmission of severe acute respiratory syndrome coronavirus 2 (SARS-CoV-2), such as travel and contact restrictions, use of personal protective equipment, and hand and cough hygiene, probably also reduced the transmission of other infectious diseases that are transmitted via direct human-to-human contact.

Besides a decrease in transmission, there may have been under-detection for some diseases, as healthcare services were focused on COVID-19 diagnosis and treatment [1-3]. An increasing body of evidence also shows that fewer people sought healthcare during the pandemic, particularly during the first wave [12-16]. According to Ullrich et al., the decrease in incidence of primarily food-borne diseases in Germany in early 2020, such as *Campylobacter* enteritis and enterohaemorrhagic *Escherichia coli* (EHEC) disease, may partly be explained by missed diagnoses due to patients not seeking care as early as they might have done before the pandemic [11].

Since 2020, the COVID-19 public healthcare burden and NPIs to prevent transmission have changed continuously. In Bavaria, a federal state in Germany with more than 13 million inhabitants, the SARS-CoV-2 Alpha and Delta variants of concern emerged, respectively, in January and May 2021, leading to higher peaks in COVID-19 case numbers than there were in 2020 [17]. Simultaneously, COVID-19 vaccines were introduced in late December 2020 and had reached a coverage of 70% of the population by the end of 2021 [17]. NPIs were adjusted in response to these developments, including a generalised lockdown in December 2020 and January 2021 involving, for example, stay-at-home orders, curfews, contact restrictions and school closures [18,19], a mandate to wear FFP2 masks in public indoor spaces from January 2021 [20], and restrictions specifically targeting the not yet immunised population from September 2021 [21]. Supplementary Table S1 provides an overview of relevant NPIs and public health events in 2020 and 2021 in Bavaria, Germany. It is unclear to what extent these dynamics have had an impact on the transmission, diagnosis and reporting of other notifiable diseases.

In order to adjust public health measures and surveillance in an adequate and timely way, it is important to continuously monitor notifiable infectious diseases in relation to the COVID-19 pandemic. Certain observations might have the following interpretations: (i) Reduced transmission of other notifiable diseases were a consequence of the efficacy of the COVID-19 NPIs; or (ii) decreases in healthcare-seeking behaviour, diagnoses or disease reporting were a consequence of a disruption in healthcare. Both scenarios may lead to a post-pandemic increase in the public health burden.

By analysing the reported cases and their characteristics during the pandemic, in comparison to the pre-pandemic phase, this study aimed to determine the impact of the pandemic on the transmission and diagnosis of other diseases in Bavaria, Germany. With our study, we hope to contribute to a better understanding and interpretation of the epidemiology of infectious diseases other than COVID-19 during and after the COVID-19 pandemic.

## Methods

### Design and setting

We analysed data from the surveillance reporting system for notifiable infectious diseases in Bavaria, Germany, at the Bavarian Health and Food Safety Authority (LGL). In Germany, clinicians and laboratories report cases of certain infectious diseases to local health authorities in accordance with the German Protection Against Infection Act §6 and §7, respectively [22]. Local health authorities forward reported cases to the LGL within 24 h. From the LGL, notifications are forwarded to the German national public health institute (Robert Koch Institute (RKI)).

According to the German Protection against Infection Act, clinicians are obliged to notify suspected and confirmed cases and fatalities of a list of diseases specified in §6. These diseases were selected based on their severity, lethality, the risk of spread in the population and the need for action by public health authorities; the list includes for example chickenpox, COVID-19 and pertussis. General practitioners, hospitals, pathological-anatomical facilities, etc. are obliged to notify. According to the German Protection against Infection Act §7, laboratories must report more than 60 pathogens if detection indicates acute infection. Detection of *Treponema pallidum*, HIV, *Echinococcus* sp., *Plasmodium* sp., *Toxoplasma gondii*, *Neisseria gonorrhoeae* and *Chlamydia trachomatis* is not reported to the local health offices, but directly and anonymously to the RKI. The German Protection against Infection Act also states which information is required in the notification and urges notifying clinicians and laboratories to provide that information to the local health authorities (§§8–12). Relevant case information includes age, gender, date of disease onset and diagnosis as well as hospitalisation information.

For this study, we included cases reported by 1 March 2022 with notification date between 1 January 2016 and 31 December 2021. We excluded diseases with less than 100 cases reported per year in at least one year between 1 January 2016 and 31 December 2019. We categorised the included notifiable diseases according to their main mode of transmission; an overview of diseases by main mode of transmission as well as annual case numbers between 2016 and 2021 is appended in Supplementary Table S2. We included at least one disease per mode of transmission, considering public health relevance in Bavaria, Germany. Table 1 shows the included diseases by main mode of transmission.

**Table 1:** Main mode of transmission of notifiable infectious diseases included, Bavaria, Germany, 2016 to 2021 (n = 353,956)

Main mode of transmission	Diseases included
Aerosols of contaminated water	Legionellosis
Airborne	Chickenpox, *Haemophilus influenzae* invasive disease
Blood-borne	Hepatitis C
Contaminated food or water	*Campylobacter* enteritis, enterohaemorrhagic *Escherichia coli* disease, salmonellosis
Droplets	Seasonal influenza, pertussis
Faecal-oral	Norovirus gastroenteritis, rotavirus gastroenteritis
Sexually transmitted and via body fluids	Hepatitis B
Vector-borne, endemic	Lyme disease, tick-borne encephalitis
Vector-borne, travel-associated	Dengue fever

### Definitions and variables

We compared weekly case numbers and case characteristics in a pre-pandemic period (1 Jan 2016–1 Mar 2020), pandemic year 1 (2 Mar 2020–31 Dec 2020) and pandemic year 2 (1 Jan 2021–31 Dec 2021). We chose 2 March 2020 as the starting date for pandemic year 1, as the start of the first COVID-19 wave in Germany was retrospectively defined to be week 10 2020 [23]. We assessed age, time-to-diagnosis and hospitalisation. Time-to-diagnosis was defined as time span in days between the date of symptom onset and the date of diagnosis. Time-to-diagnosis was only determined for symptomatic cases for whom information on date of onset and diagnosis was available and where disease onset was before diagnosis.

### Data analysis

For each disease investigated, we first performed a descriptive analysis of the cases included in the study, including year of notification, age, gender and hospitalisation as indication of the severity of disease.

### Change of weekly case numbers during the pandemic

We conducted a time series analysis for each disease based on the weekly aggregated number of cases to assess weekly notifications of the diseases included during the COVID-19 pandemic compared with the pre-pandemic period.

In a first analysis, we generated a predictive model based on the weekly case numbers from 2016 to 2019 using a negative binomial regression, including trend and seasonality. We examined whether there was a trend over the period 2016 to 2019 using univariable negative binomial regression to assess the association between time (week and year) and weekly aggregated case numbers. Periodicity was defined based on literature, supported by the assessment of disease-specific periodograms for weekly case numbers in 2016 to 2019 [24]. Seasonality was accounted for in the negative binomial regression model by incorporating corresponding sine and cosine terms. Where applicable, we also considered changes in case definitions by including a variable for the periods in which the respective definitions applied. Based on this predictive model, we determined expected weekly case numbers for 2020 and 2021, including respective 95% prediction intervals. We compared these with the observed number of cases.

To quantify observed effects, we included in a second analysis the pandemic periods (pre-pandemic, pandemic year 1, pandemic year 2) as a variable in the models and determined respective incidence rate ratios (IRR) and confidence intervals (CI).

### Change of case characteristics during the pandemic

We determined median age, median time-to-diagnosis, proportion hospitalised and corresponding data completeness (proportions of cases for whom information for each of the included characteristics was available) for the pre-pandemic period, pandemic year 1 and pandemic year 2. We used univariable logistic regression to compare data completeness (age, time-to-diagnosis and hospitalisation) and hospitalisation rates in the different periods. We used the Wilcoxon rank-sum test to compare the median age and time-to-diagnosis. All data analyses were performed in R (Version 4.0.2; Vienna, Austria: R Foundation for Statistical Computing), using the tidyverse, MASS and trending packages.

## Results

From 2016 to 2021, 1,709,659 cases of notifiable diseases were reported in Bavaria, Germany. We included 353,956 cases of the selected diseases in our analyses. Of those, 267,258 cases (76%) were reported by laboratories, 51,319 (14%) by physicians, 17,636 (5%) by other entities and for 17,743 (5%), no information on the notifying entity was available. Case numbers and characteristics for each disease are provided in Supplementary Table S3.

### Change of weekly case numbers during the pandemic

**Figure 1:**
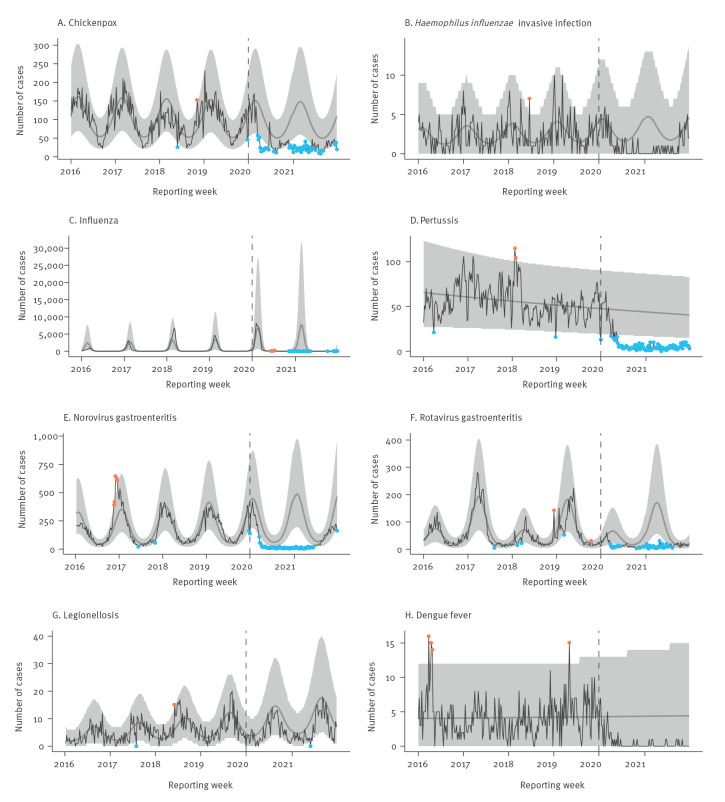
Cases of selected respiratory, gastro-intestinal and travel-associated diseases by reporting week, Bavaria, Germany, 2016–2021 (n = 253,596) plotted against predicted weekly case numbers based on 2016–2019 notifications

**Figure 2:**
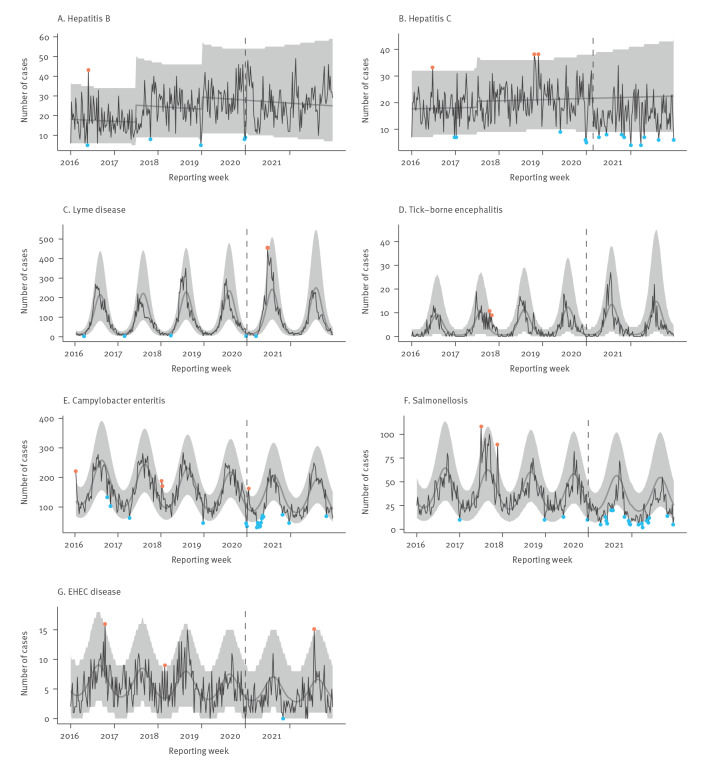
Cases of selected sexually transmitted, blood-, endemic vector- and food-borne diseases by reporting week, Bavaria, Germany, 2016–2021 (n = 100,360) plotted against predicted weekly case numbers based on 2016–2019 notifications

In Figures 1 and 2, observed case numbers per disease for 2016 to 2021 are plotted against predicted case numbers based on the data from 2016 to 2019. Table 2 contains case numbers by period and respective IRR. For all diseases assessed, except for Lyme disease, TBE and influenza, there were significantly fewer cases in pandemic year 1 than in the pre-pandemic period. We observed the largest reduction in case numbers for norovirus gastroenteritis (IRR = 0.15; 95% CI: 0.12–0.20), dengue fever (IRR = 0.13; 95% CI: 0.08–0.21) and pertussis (IRR = 0.22; 95% CI: 0.18–0.26).

Black line: reported cases; grey line: predicted cases; shaded areas: 95% prediction intervals. Blue dots: weeks in which case numbers were below the 95% prediction interval; orange dots: weeks in which case numbers were above the 95% prediction interval.

EHEC: enterohaemorrhagic *Escherichia coli*.

Black line: reported cases; grey line: predicted cases; shaded areas: 95% prediction intervals. Blue dots: weeks in which case numbers were below the 95% prediction interval; orange dots: weeks in which case numbers were above the 95% prediction interval.

**Table 2:** Cases of selected notifiable infectious diseases reported and respective incidence rate ratios, by period, 2016–2021, Bavaria, Germany (n = 353,956)

Disease and period	Number of cases	IRR (95% CI)	p-value
***Campylobacter* enteritis**			
Pre-pandemic (yearly average)	34,313 (8,355)	Reference	
Pandemic year 1	5,017	0.72 (0.65–0.79)	< 0.001
Pandemic year 2	6,609	0.91 (0.81–1.02)	0.095
**Chickenpox**			
Pre-pandemic (yearly average)	21,439 (5,079)	Reference	
Pandemic year 1	1,487	0.41 (0.35–0.49)	< 0.001
Pandemic year 2	1,352	0.31 (0.26–0.38)	< 0.001
**Dengue fever**			
Pre-pandemic (yearly average)	893 (217)	Reference	
Pandemic year 1	23	0.13 (0.08–0.21)	< 0.001
Pandemic year 2	9	0.04 (0.02–0.08)	< 0.001
**EHEC disease**			
Pre-pandemic	1,195 (294)	Reference	
Pandemic year 1	163	0.75 (0.60–0.93)	0.009
Pandemic year 2	207	0.90 (0.71–1.15)	0.415
***Haemophilus influenzae*, invasive infection**			
Pre-pandemic (yearly average)	538 (126)	Reference	
Pandemic year 1	38	0.34 (0.23–0.51)	< 0.001
Pandemic year 2	47	0.30 (0.19–0.45)	< 0.001
**Hepatitis B**			
Pre-pandemic (yearly average)	5,065 (1,189)	Reference	
Pandemic year 1	970	0.73 (0.62–0.86)	< 0.001
Pandemic year 2	1,472	0.90 (0.71–1.13)	0.364
**Hepatitis C**			
Pre-pandemic (yearly average)	4,327 (1,037)	Reference	
Pandemic year 1	664	0.70 (0.60–0.81)	< 0.001
Pandemic year 2	820	0.70 (0.58–0.85)	< 0.001
**Influenza**			
Pre-pandemic (yearly average)	140,742 (25,270)	Reference	
Pandemic year 1	14,453	1.35 (0.65–2.73)	0.352
Pandemic year 2	153	0.04 (0.02–0.09)	< 0.001
**Legionellosis**			
Pre-pandemic (yearly average)	1,185 (289)	Reference	
Pandemic year 1	252	0.63 (0.50–0.76)	< 0.001
Pandemic year 2	338	0.65 (0.50–0.83)	< 0.001
**Lyme disease**			
Pre-pandemic (yearly average)	17,636 (4,376)	Reference	
Pandemic year 1	6,130	1.20 (1.02–1.42)	0.03
Pandemic year 2	3,995	0.82 (0.68–0.99)	0.04
**Norovirus gastroenteritis**			
Pre-pandemic (yearly average)	39,079 (9,105)	Reference	
Pandemic year 1	1,076	0.15 (0.12–0.20)	< 0.001
Pandemic year 2	3,572	0.40 (0.29–0.55)	< 0.001
**Pertussis**			
Pre-pandemic (yearly average)	12,141 (2,929)	Reference	
Pandemic year 1	422	0.22 (0.18–0.26)	< 0.001
Pandemic year 2	235	0.11 (0.09–0.14)	< 0.001
**Rotavirus gastroenteritis**			
Pre-pandemic (yearly average)	12,953 (3,157)	Reference	
Pandemic year 1	496	0.35 (0.26–0.47)	< 0.001
Pandemic year 2	673	0.34 (0.26–0.45)	< 0.001
**Salmonellosis**			
Pre-pandemic (yearly average)	8,310 (2,028)	Reference	
Pandemic year 1	1,049	0.60 (0.52–0.70)	< 0.001
Pandemic year 2	1,131	0.61 (0.52–0.72)	< 0.001
**Tick-borne encephalitis**			
Pre-pandemic (yearly average)	820 (205)	Reference	
Pandemic year 1	280	1.08 (0.80–1.47)	0.60
Pandemic year 2	187	0.68 (0.46–1.00)	0.04

CI: confidence interval; EHEC: enterohaemorrhagic *Escherichia coli*; IRR: incidence rate ratio.

Pre-pandemic = week 1 of 2016 to week 9 of 2020; pandemic year 1 = weeks 10 to 52 of 2020; pandemic year 2 = weeks 1 to 52 of 2021.

In 79% of the weeks in 2020, norovirus gastroenteritis weekly case numbers were below the 95% prediction interval; in weeks 26–50 of 2020, only two dengue fever cases were reported. Pertussis weekly case numbers were below the 95% prediction interval in weeks 20–50 of 2020. Weekly case numbers for chickenpox, rotavirus gastroenteritis and salmonellosis were below the 95% prediction interval in 38%, 35% and 19% of the weeks in 2020, respectively. *Campylobacter* enteritis weekly case numbers were below the 95% prediction interval in weeks 1, 13–21, 44 and 52 of 2020. Weekly case numbers of legionellosis, hepatitis C and B, *Haemophilus influenzae* invasive disease and EHEC disease were never or rarely below the 95% prediction interval in 2020. However, in 83%, 81%, 63%, 75% and 67% of the weeks, weekly case numbers for these five diseases, respectively, were below the average predicted case numbers based on data for 2016 to 2019. In contrast to all other diseases assessed, we observed an increase in the weekly cases of Lyme disease in pandemic year 1 (IRR = 1.20; 95% CI: 1.02–1.42).

In pandemic year 2, there were significantly fewer cases for all assessed diseases except for Lyme disease, TBE, *Campylobacter* enteritis, EHEC disease and hepatitis B. Influenza, dengue fever and pertussis case numbers decreased most markedly (IRR = 0.04 (95% CI: 0.02–0.09); IRR = 0.04 (95% CI: 0.02–0.08); IRR = 0.11 (95% CI: 0.09–0.14), respectively). In the pre-pandemic period, there were between one and 6,816 cases of influenza per week. Between week 28 of 2020 and week 52 of 2021, there were fewer than 20 cases of influenza per week. Pertussis weekly case numbers were below the 95% prediction interval in all weeks in 2021. Weekly rotavirus gastroenteritis, norovirus gastroenteritis and chickenpox case numbers were below the 95% prediction interval until week 28, 24 and 22 in 2021, respectively.

### Change of case characteristics during the pandemic

Detailed case characteristics per period are appended in Supplementary Table S4. Most changes in case characteristic were observed for EHEC disease: There was a decrease in the median age of EHEC disease cases in pandemic years 1 (4 years; interquartile range (IQR): 1–32) and 2 (3 years; IQR: 1–18) compared with the pre-pandemic period (11 years; IQR: 2–42). The proportion of 16–59-year-old cases decreased by 50% in pandemic year 1 (34% vs 18%). The proportions of cases younger than 16 years, and 60 years and older, increased (54% vs 67% and 12% vs 15%, respectively). For a detailed overview of EHEC cases and hospitalisations by age group see Supplementary Table S5. There was an increase in the proportion of hospitalised EHEC disease cases in pandemic year 1 (43/148, 29%) compared with the pre-pandemic period (220/1,082, 20%; odds ratio = 1.60; 95% CI: 1.08–2.34) (Supplementary Table S4). The proportion of hospitalised EHEC disease cases 60 years and older almost doubled in pandemic year 1 (36% vs 68%) (Supplementary Table S5).

There were also changes in the median age of influenza, norovirus gastroenteritis and pertussis cases: The median age of influenza cases decreased in pandemic year 1 (30 years; IQR: 11–51) compared with the pre-pandemic period (38 years; IQR: 13–57). The median age of norovirus gastroenteritis cases increased in pandemic year 1 (56 years; IQR: 21–79) and decreased in pandemic year 2 (27 years; IQR: 2–62) compared with the pre-pandemic period (50 years; IQR: 15–77), with median age ranging from 38 years (IQR: 8–73) in 2016 to 53 years (IQR: 20–78) in 2017. The median age of pertussis cases increased in pandemic year 1 (43 years; IQR: 17–60) and in pandemic year 2 (48 years; IQR: 30–65) compared with the pre-pandemic period (38 years; IQR: 14–55).

There was no relevant change in time-to-diagnosis for the included diseases. The quality of hospitalisation data decreased for all diseases in pandemic year 1 and 2 except for dengue fever, EHEC disease, *H. influenzae* invasive disease, legionellosis, pertussis and TBE, hampering the assessment of changes in disease severity based on hospitalisation data.

## Discussion

The study shows that during the COVID-19 pandemic, particularly in 2020 and to a lesser extent also in 2021, there were significantly fewer cases of notifiable diseases other than COVID-19. There were also shifts in case characteristics, such as a decrease in median age and an increase in hospitalisations for EHEC disease. The reasons for these observations are likely to be multifactorial, and our analyses of surveillance data cannot determine with certainty to which extent decreases in disease reporting were due to a true decrease in transmission or whether the decrease was only due to a decrease in diagnosis. However, by assessing diseases with different modes of transmission and examining case characteristics, we can derive hypotheses regarding the reasons for the reduction in case numbers.

There may have been a decrease in transmission, and thus a true decrease of cases, for several notifiable diseases as a consequence of the COVID-19 NPIs as indicated by the decrease in weekly chickenpox, *H. influenzae* invasive infection, pertussis, norovirus and rotavirus gastroenteritis cases in pandemic years 1 and 2 and influenza cases in pandemic year 2. These diseases are mainly transmitted via direct close contact, i.e. airborne, via droplets and faecal-oral transmission. Although in our study the case numbers for influenza in pandemic year 1 were similar to previous years, national analyses show that the 2019/20 influenza wave lasted only 11 weeks and thus was shorter than the five previous seasons (13–15 weeks) [25]. Hence, as described before, COVID-19 NPIs such as increased hand and cough hygiene, physical distancing, contact restrictions and the closure of schools and daycare centres, probably also reduced infections caused by transmission via direct close contact [4].

We also observed a significant decrease in the median age of norovirus cases in 2021. This shift possibly reflects an increase in cases, particularly among younger children, after the discontinuation of NPIs (e.g. schools gradually re-opened after March 2021). Similar effects have already been observed in other studies for RSV, rhino-/enterovirus, norovirus and influenza A and B in various settings [26-28]. One hypothesis is that a reduction of exposure to pathogens during NPIs led to decreased community immunity, particularly in children [4]. Thus, although we observed continued decreases in influenza and norovirus case numbers in 2021, it will be important to further monitor these trends to see if there is a resurgence of infections after the end of the NPIs.

The significant decrease in legionellosis and dengue fever cases in pandemic years 1 and 2 may be an effect of COVID-19 NPIs, particularly travel restrictions. International travel was associated with 100% of dengue fever cases and 23% of legionellosis cases in 2019 [29], suggesting that travel restrictions imposed to reduce COVID-19 transmission probably affected transmission of these diseases, too. Interestingly, the trend of fewer infections continued in 2021 when travel was increasing again. Both legally enforced restrictions and changes in travel behaviour while legal restrictions were in place, as well as after they were lifted, may have played a role. A study found that in Germany, fewer people travelled abroad for holidays in 2020 than in 2019, while domestic travel increased over the same period [30]. In 2021, the number of trips abroad increased compared with 2020 but were still below the levels of 2019. A change in healthcare-seeking behaviour is probably not a sufficient explanation for the observed reduction in case numbers, as almost all dengue fever cases are hospitalised, as well as legionellosis cases, who (by definition) suffer from pneumonia.

The decrease in weekly case numbers of the primarily food-borne diseases EHEC disease and *Campylobacter* enteritis in pandemic year 1 and salmonellosis in both years indicate that diagnosis and healthcare-seeking was reduced during the pandemic. In Germany, most EHEC cases younger than 3 years are associated with prior contact to ruminants, consumption of raw milk, or a person with diarrhoea in the family; for cases older than 9 years, transmission is primarily food-associated [31]. *Campylobacter* enteritis is often associated with consumption of meat and raw milk [32]. Salmonellosis is most often caused by the consumption of raw or poorly cooked eggs and meat [33]. To a certain extent, the reduction may be explained by contact restrictions reducing the possible risk of exposure at e.g. petting zoos, restaurants and public events. However, the risk of exposure to food-borne pathogens in the individual households remained. According to the European Food Safety Authority, most food-borne outbreaks are associated with exposures on domestic premises [34]. Thus, also decreased healthcare-seeking behaviour may have played a role. This hypothesis is supported by the observed decrease in median age of EHEC disease cases and simultaneous increase in the proportion of hospitalisations in pandemic year 1, particularly among patients 60 years and older. Infants and elderly people are more likely to develop severe EHEC disease, and EHEC disease incidence in Germany is highest among children younger than 5 years [31]. Hence, it is possible that mainly young EHEC patients at risk of severe disease and elderly patients with severe symptoms that required hospitalisation sought care during the beginning of the pandemic. In 2021, case numbers recovered to pre-pandemic levels. Relaxed contact restrictions from March 2021 as well as reduced risk perception in the population [35] could have led to renewed healthcare-seeking behaviour, which may partly explain this observation.

We observed an increase in Lyme disease cases in pandemic year 1, similar to the increase in TBE observed in Germany early 2020 and possibly linked to increased outdoor activities in 2020 [11]. This may indicate consistent healthcare-seeking behaviour for this disease, which would not be surprising considering the characteristic symptoms (erythema migrans) and possible severity of disease if not treated.

Multiple factors, including under-detection, may have played a role in the decrease of weekly hepatitis B and C case numbers in pandemic year 1, and, for hepatitis C, in pandemic year 2. Most incident hepatitis B cases in Germany are likely to be caused through sexual transmission. The disease is common among migrants from high-incidence countries, sex workers, men who have sex with men (MSM) and persons who use injection drugs [29,36]. A study from Belgium showed a significant decrease in sex with casual partners among MSM during the first weeks of the lockdown in 2020 [37]. Thus, to a certain degree, the observed decrease in hepatitis B case numbers may be due to contact restrictions which possibly also led to reduced sexual contacts. Hepatitis C risk groups are migrants from high incidence countries, persons who use injection drugs, and persons in detainment [38]. In 2019, almost 50% of the hepatitis B cases and 20% of hepatitis C cases notified in Germany, for whom information on the country of exposure was available, contracted the infection abroad [29]. Consequently, decreases in travel and migration during the pandemic may have contributed to decreased case numbers of hepatitis B and C. The decrease in hepatitis B and C case numbers may have been caused in part by under-detection as low-level testing facilities were closed or had reduced opening hours. A reduction in hepatitis B and C testing rates was observed in several European countries in 2020 [39]. Lack of adequate and early testing and treatment may also have led to an as-yet undetected increase of hepatitis B and C transmission. It is unclear why the negative trend in case numbers continued in 2021 only for hepatitis C and not hepatitis B. Considering that particularly hard-to-reach and vulnerable populations are affected by hepatitis C, and that delayed diagnosis and treatment may lead to severe consequences of disease such as liver cirrhosis, it will be important to monitor this development and to further assess whether the decrease is truly caused by under-detection.

This study has several limitations. Firstly, the assessment of just three time periods (pre-pandemic, pandemic year 1 and pandemic year 2) represents a simplified approach of assessing effects of the pandemic. During the two pandemic periods, there were various, sometimes overlapping COVID-19-related developments, such as: new variants, newly introduced vaccines or vaccination recommendations, new NPIs and changing perception of the pandemic in the population [35]. Probably all of these developments had different impacts on the healthcare system and on healthcare-seeking behaviour, and consequently on the diagnosis and notification of other infectious diseases. As it is difficult to disentangle these effects, we chose the pragmatic approach of assessing three different periods. Results from these analyses were interpreted with careful consideration of weekly case numbers and characteristics.

Secondly, not for all diseases a well-fitting time series model could be established as this would have required parameters not available in surveillance data. However, the applied models still facilitate conclusions regarding general trends.

Finally, quality and completeness of surveillance data regarding the time-to-diagnosis and hospitalisation were limited. Therefore, for some diseases, no comparison of time-to-diagnosis and hospitalisation could be made between the considered periods. This finding, however, may be valuable, considering the increased workload that the pandemic caused for public health services.

## Conclusion

COVID-19 NPIs seem to have been successful in preventing transmission of various infectious diseases, especially during the pandemic in 2020, and to a lesser extent in 2021. However, during the pandemic, and particularly in 2020, people may have sought healthcare and diagnosis less frequently. As insights into healthcare-seeking behaviour cannot be drawn from the analysis of surveillance data, further research is needed to identify changes in access to healthcare due to the pandemic, as well as potential underlying causes and countermeasures. Further monitoring of epidemiological trends will be needed to identify if, for example, there will be a post-NPI resurgence of case numbers due to a higher proportion of susceptible persons in the population, or an increase in healthcare burden due to delayed diagnosis of certain diseases. Our analyses provide baseline data as well as a methodology for monitoring and may aid in the interpretation of such trends in the future.

## Supplementary Data

60000 Supplement
